# Lateral Position Measurement Based on Vehicles’ Longitudinal Displacement

**DOI:** 10.3390/s20247183

**Published:** 2020-12-15

**Authors:** Ibrahim Mohsen, Thierry Ditchi, Stéphane Holé, Emmanuel Géron

**Affiliations:** 1 LPEM, PSL Université, ESPCI-Paris, CNRS, 10 rue Vauquelin, 75005 Paris, France; ibrahim.mohsen@espci.fr; 2 LPEM, Sorbonne Université, CNRS, 10 rue Vauquelin, 75005 Paris, France; thierry.ditchi@espci.fr (T.D.); stephane.hole@espci.fr (S.H.)

**Keywords:** lateral positioning sensor, phase difference of arrival (PDOA), vehicle positioning, cooperative electromagnetic systems, advanced driving assistance system (ADAS)

## Abstract

The lateral position of a vehicle in its lane is crucial information required to develop intelligent assistant driving systems. Current studies reveal this information by mixing multiple sources such as cameras, LiDAR or accurate GNSS. Because these systems are not efficient in some degraded weather conditions, a cooperative Vehicle-to-Infrastructure sensor has been developed to help to determine lateral position of a vehicle in its lane. In this paper, the authors propose a completely new and original way to estimate lateral position of the vehicle in its lane using the longitudinal displacement. Using a system based on a hyper-frequency interaction between a transceiver module embedded in the vehicle and passive transponders that can be integrated in the road, for instance under the lane markings, a new signal processing algorithm is presented in order to determine the lateral distance between the vehicle and the transponder axis. The sensor has been tested in an external environment and has shown an estimated lateral distance error of 8 cm at most.

## 1. Introduction

Autonomous vehicles are the heart of Intelligent Transportation Systems (ITS). ITS aims to reduce vehicle crashes and road fatalities, to optimize trajectory duration, to decrease pollution and to give more comfort to passengers. More information are needed to ensure a proper function of the ITS like traffic information, weather information, vehicle global positioning and of course precise local positioning in its own road lane. In fact, autonomous systems rely on external signs, such as the road edges, markings, satellites, panels and so on. In this trend, communications between vehicles and between vehicles and infrastructure are an essential key to reach such goals. In the case of autonomous vehicles, many positioning systems have been developed using different techniques such as satellite navigation system, computer vision, map assisted system, LiDAR and Vehicle-to-Infrastructure (V2I) systems based on integrated sensors in the road [1,2].

Global Navigation Satellite System (GNSS) is considered as the most commonly used system to find the vehicle global position. Several studies use GNSS signal combined with other sensors, such as Inertial Navigation System (INS), vehicle motion sensors, digital road maps or laser scanners, in order to refine the accuracy of GNSS and to reach the requested precision for autonomous vehicle applications [3,4,5,6,7,8]. However, GNSS signal is widely affected by buildings, trees and any other elements that deflect the direct path of electromagnetic waves from satellites to the vehicle. None of existing devices can supply the required precision in real-time in all situations. Vision systems based on cameras currently detect road markings to find the vehicle position in its lane using digital maps to increase the accuracy of vehicle positioning systems [9,10,11]. All these systems are accurate when road markings are clear and environmental conditions are suitable. During the last years, vision systems with different approaches for movement detection [12] have made great progresses in detection even in bad conditions using splay angle or optical flow techniques for example. However, these techniques always need the detection of a marking lane or point of interest like a building edge in the environment [13,14]. So, in some conditions like nighttime luminosity, bad weather (intensive rain or snow) or when road markings are overused or simply obstructed, the efficiency of these systems is deteriorated [15,16,17]. LiDAR is another vision system that generates a 3D map of the road and of the surrounding which is matched with already known data in order to localize the vehicle [18,19]. Unfortunately, this technique is still expensive and perturbed in case of intensive rain, fog and in presence of other vehicles which can mask the environment. Some optimized LiDAR systems are studied to involve detection in fog with good perspectives but these systems are laboratory prototypes limited to less than one meter in front detection [20]. So LiDAR cannot be used immediately for large commercial application. Particularity of all the systems discussed above is that they rely only on the existing infrastructure.

One can consider another kind of positioning system that requires specific infrastructure installations which is known as Vehicle-to-Infrastructure (V2I) systems. These systems use many kinds of communication between vehicle and infrastructure in order for instance to locate the vehicle on road. This is part of the fifth infrastructure generation development [21].

In the case of positioning, some of these V2I systems use magnets burred in or deposited on the road so they are mostly not disturbed by weather conditions [22,23,24]. However, magnetic field is inversely proportional to the cube of distance between magnet and sensor [25], so an installation at the lane edge or underneath the surface implies the use of strong magnets to cover all the roadway. Therefore these systems are very expensive to install and their large scale implementation is likely impossible.

Active RFID positioning [26] scheme is another solution. While passing above tags, the reader in the vehicle gets the tag information on road that contains the local position. Currently only theoretical aspects are presented without experimental results.

Some other V2I systems using active electromagnetic landmarks have been studied. In [27], the Doppler effect is used from two radio beacons. It has been demonstrated in real conditions, that the lane can be detected from the frequency offset (CFO). However, the limited precision of the system (10 cm at best), is not sufficient for in lane positioning. In [28], a specific numerical process is used to determine the lateral and longitudinal positions of the vehicle. Here again the position accuracy (about 28 cm) is not suitable for lateral positioning. These two systems are quite expensive to install and may generate excessive electromagnetic pollution. According to the authors, they may only be used at strategic positions, for instance at crossroads where GNSS has a poor coverage, but not for lateral positioning along a road.

In the future, V2I and V2V communications will extend their contributions and many systems will be included in vehicles in order to inform or be informed by other vehicles or static bases about traffic flow, accident, road weather conditions, vehicle integrity, all useful information for drivers. In [29] an adaptative algorithm based on an Extended Kalman filter (EKF) is proposed to fuse data from fixed or mobile sources in order to improve localization of vehicle on the road. Using the angle of arrival of beacon received packets, the position of the vehicle in its lane is greatly improved. However, except accuracy, this system suffers from the same limitations as the two former ones, its price, and thus could be installed only in strategic areas. We can note that according to the authors of this paper, few precise localization measurements that can be provided for example by our new localization sensor we present here after, even if it is implemented in few vehicles, can contribute greatly to the performance of localization of a lot of vehicles in an urban area or in dense traffic.

To overcome these difficulties and to guaranty a low cost system only compatible with a large scale deployment, a functional prototype of a V2I sensor based on a high-frequency interaction was presented and described in [30,31,32]. The system operate in the Ultra High Frequency (UHF) band to provide a large penetration depth in water and snow. It has therefore a great immunity against most weather conditions. As all existing sensors, it is intended to be coupled with other existing sensors to improve security and accuracy of Driving Assistant System.

The sensor described in [31,32] is composed of two parts: the first part is a high-frequency transceiver present on each side of the vehicle and the second part is a set of passive transponders sufficiently flat to be integrated in the lane markings. Based on the phase of the back-scattered signal from the transponders that are excited by the embedded transceiver, round trip distance between transmitting and receiving antenna on the vehicle is determined. Assuming a transponder placed on the perpendicular bisector of the segment joining emitting and receiving antennas, the distance between vehicle and lane marking is then calculated with an accuracy of ±3 cm. Papers [31,32] validated the use of two resonant frequencies to avoid phases wrapping in $\left[-\pi ,\pi \right]$ range.

In the present paper, a completely different algorithm allows the determination of distance between vehicle and lane marking without any assumption on vehicle position with respect to transponders and with only one resonator embedded into transponders. The aim of Section 2 is to explain this new algorithm. In Section 3, the results obtained from experimental measurements done in quasi-static conditions and simulations are shown in order to evaluate the robustness of the algorithm assuming vehicle longitudinal and lateral accelerations during measurements. In the last section before the conclusion, different issues are discussed about possible spurious sources, the electromagnetic interference between vehicles, cost and security.

## 2. Algorithm Principle

**Figure 1:**
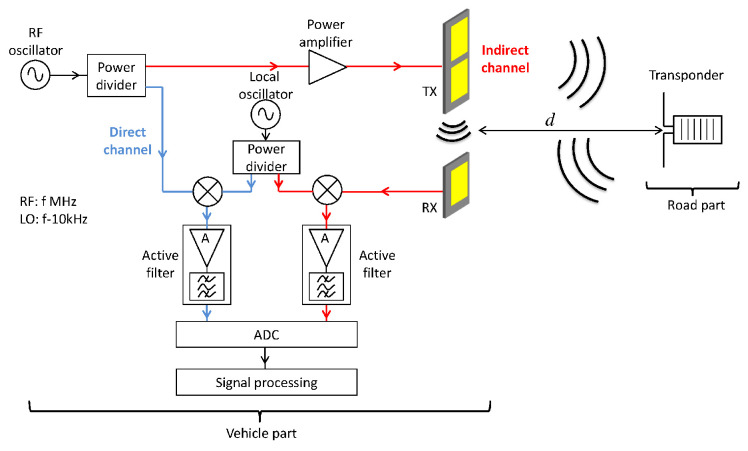
Block diagram of the lateral positioning system.

The sensor hardware is similar to the one described in reference [32]. The block diagram of the lateral positioning system is recalled in Figure 1. It is composed of two parts, one on the road embedded in white marking and the other in the vehicle.

The road part of the system consists of passive transponders. Each one is composed of a flat half-wave antenna connected to a Surface Acoustic Wave (SAW) resonator in order to distinguish the reflected weak signal from other spurious signals. The SAWresonator is a key compound of the transponders because it ensures to unambiguously detect transponders from the background noise. These resonators are also very common, available in quantity and at low price. As the half-wave antennas are simply 2 wires, the overall price of a transponder is in the range of cents.

The vehicle part consists of an hyper-frequency emission-reception module with two patch antennas. In a sense, it is similar to an anti-collision radar system, except that it works at lower frequencies and for shorter distances. As conventional ISM components are used, they are available in quantity and at low price.

It is worth noting that compared to the system described in [32], just one resonator instead of two is required for transponders in the present study. The system operates around the central frequency of the SAW resonator ($f_{0}=868.3\;\mathrm{MHz}$) which is an Industrial, Scientific and Medical (ISM) radio band.

The on-board RF oscillator generates a sine wave which frequency sweeps from 867.5MHz to 869.5MHz by step of 20kHz. In this on-board RF system, there are two channels. In the direct channel, the RF signal is down-converted to an Intermediate Frequency (IF) at 10 kHz and digitized to be numerically processed. In the second channel (the indirect one) the RF signal is amplified and transmitted by the emitting antenna. In this second channel, the received signal is treated similarly to the direct channel, that is to say down-converted to the same Intermediate Frequency and digitized.

**Figure 2:**
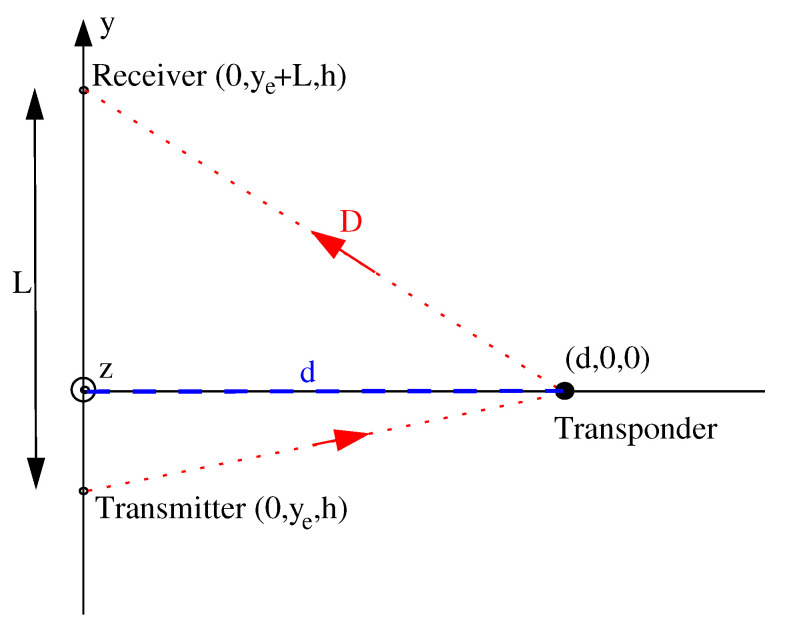
Definition of the round trip distance *D* (- - -) and the lateral distance *d* (- - -).

The reflected signal from the transponder presents a sharp resonance producing a fast variation of amplitude and phase around the central frequency ($f_{0}=868.3\;\mathrm{MHz}$) of the resonator. This particular variation, called hereafter transponder signature, is perfectly known and is exploited to extract the transponder signal from the whole received signal. Two distances have to be defined. The lateral distance *d* (see Figure 1 and Figure 2) which is the sought distance from transponder perpendicular to the vehicle side. There is also the round trip distance *D* (see Figure 2) which corresponds to the path length from the emitting antenna to the transponder then to the receiving antenna.

First of all, a signal processing extracts the signal reflected by the transponder (called transponder signal in the following) from the received signals. Extraction of the transponder signal is obtained by modeling the total received signal as a superimposition of two terms. The first term corresponds to the signal reflected by the transponder which contains the signature centered around $f_{0}$ delayed by the transit time of electromagnetic wave from the vehicle to the transponder. The second term includes all other spurious signals such as direct coupling signal between emitting and receiving antennas, reflected signals on surrounding objects, other emitters, which do not contain any transponder signature. The principle of this extraction has been reported in [32].

Contrarily to [32], the lateral distance *d* is calculated, in the present work, with a totally different process without assuming the vehicle longitudinal position with respect to transponders and using only one resonator by transponders. The algorithm uses the longitudinal displacement of the vehicle in its road lane to calculate the lateral distance from the lane marking in which transponders are embedded.

### 2.1. Round Trip Distance D Determination

Calculation of the round trip distance *D* is based on the phase of the transponder signal. In free space, this phase, for instance at resonance frequency $f_{0}$, varies as

(1) $$\varphi_{0}=-\frac{2\pi D}{\lambda_{0}}$$

where $\lambda_{0}=34.55\;\mathrm{cm}$ is the wavelength of the electromagnetic wave at frequency $f_{0}$.

As the measured phase $\varphi_{m}$ is determined with an indeterminacy of 2π, Equation (2) shows that distance *D* is determined with an indeterminacy of $n\lambda_{0}$, where n is an integer.

$$\varphi_{m}=\varphi_{0}+n2\pi$$

(2) $$D=D_{m}+n\lambda_{0}\;\mathrm{with}\;D_{m}=-\frac{\varphi_{m}}{2\pi }\lambda_{0}$$

In [32], the absolute round trip distance *D* is calculated using SAW with two resonant frequencies to determine the value of *n*. However in the present paper, *n* has no longer to be determined thanks to the evolution of $D_{m}$ during the vehicle displacement.

### 2.2. Lateral Distance D Determination

As the relation between *D* and *d* depends on the position of the vehicle with respect to the transponder, lateral distance *d* cannot be estimated based on only one measurement. A set of measurements must be done before calculating the lateral distance. In using Cartesian coordinates shown in Figure 2 where the transponder is located on the x-axis, and the vehicle displacement is along y-axis, distance D(t) can be expressed as a function of time t during the displacement of the vehicle can be written as

(3) $$D\left(t\right)=\sqrt{d{\left(t\right)}^{2}+y_{e}\left(t\right){}^{2}+h^{2}}+\sqrt{d{\left(t\right)}^{2}+{\left(y_{e}\left(t\right)+L\right)}^{2}+h^{2}},$$

where the emitting antenna ordinate $y_{e}\left(t\right)=y_{e}\left(0\right)+v_{Long}\left(t\right)\;t$ and $d\left(t\right)=d\left(0\right)+v_{Lat}\left(t\right)\;t$. In this equation, both the distance *L* between on-board antennas and the height *h* of on-board antennas are known. The vehicle moves along *y*-axis with a longitudinal velocity $v_{Long}$ and a lateral velocity $v_{Lat}$, and $\left(d\left(0\right),y_{e}\left(0\right)\right)$ is the position of the set of measurements at t=0.

**Figure 3:**
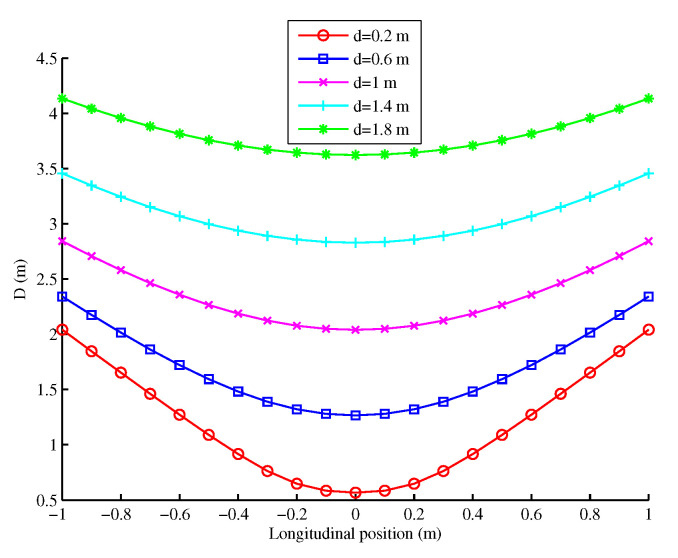
Distance *D* as a function of the longitudinal position.

Based on Equation (3), Figure 3 illustrates the distance $D\left(i\right)$ for different constant lateral distances $d\left(t\right)\;=d\left(0\right)$ in other words $v_{Lat}\;=0$ and for a constant longitudinal velocity $v_{Long}\left(t\right)\;=v_{Long}\left(0\right)$. It can be seen that each curve is characterized by a unique curvature depending on distance *d*.

In Equation (3), $d\left(0\right)$ and $y_{e}\left(0\right)$ are not known. These two unknowns can be deduced from the set of measured distances $D_{m}$ using their curvature feature. In fact, there is only one couple $\left(d\left(0\right),y_{e}\left(0\right)\right)$ that yields to the distance $D\left(t\right)$ with the same curvature as the one of the measured distance $D_{m}$. The couple $\left(d\left(0\right),y_{e}\left(0\right)\right)$ is obtained by minimizing the cost function given by Equation (4). This cost function corresponds to the standard deviation of the difference between $D_{m}$ and *D* for all longitudinal measurement positions. Minimizing this cost function ensures the best parallelism between the two curves. Once Equation (4) is minimized, the initial lateral distance $d\left(0\right)$ and the initial position $y_{e}\left(0\right)$ can be deduced. Note that cost function minimization is based on the trust region algorithm [33].

(4) $$F_{cost}=\sigma \left(D_{m}\left(i\right)-D\left(i\right)\right)=\sqrt{\frac{\sum_{0}^{N-1}{\left(\left(D_{m}\left(i\right)-D\left(i\right)\right)-\bar{\left(D_{m}\left(i\right)-D\left(i\right)\right)}\right)}^{2}}{N}}$$

where σ is the standard deviation and *N* is the number of measurements during the longitudinal displacement of the vehicle beside the transponder.

**Figure 4:**
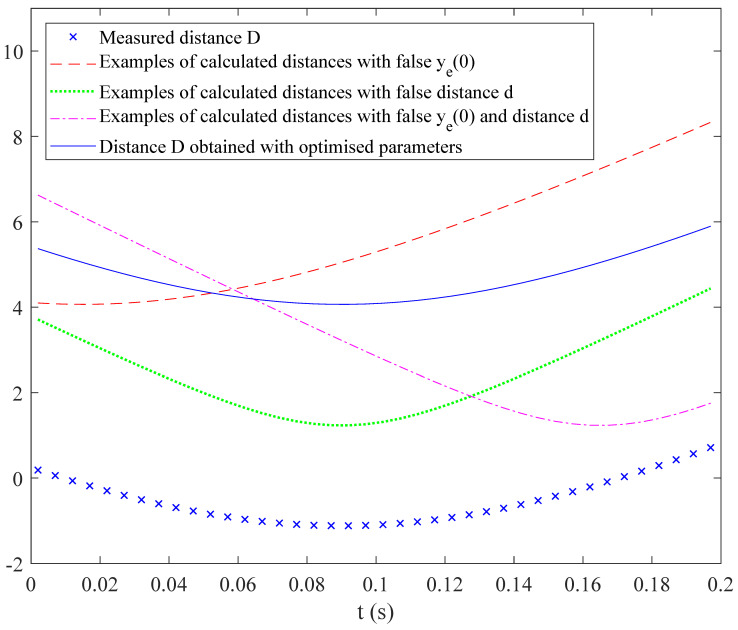
Example of a distance *D* versus time *t* during the displacement and comparison with some theoretical distance curves for various parameter couples and curve obtained after optimization of parameters.

Figure 4 shows an example of measured distance $D_{m}$ (in blue crosses). During the minimization of the cost function, various parameter couples are tried, leading to the distance *D* plotted in discontinuous lines. Red dashed line curve is obtained with false longitudinal position $y_{e}\left(0\right)$, green dotted line with false lateral distance $d\left(0\right)$, and dashed dotted magenta line with $d\left(0\right)$ and $y_{e}\left(0\right)$ false simultaneously. After optimization, the algorithm converges to the blue continuous curve. It illustrates that even with distance $D_{m}$ determined from the phase $\varphi_{m}$ with an indeterminacy of $n\lambda_{0}$, the best parallel curve is obtained with a good couple $\left(d\left(0\right),y_{e}\left(0\right)\right)$. Here, the only assumption is that the lateral distance does not vary more than one $\lambda_{0}$ between two successive measurements, which leads to a minimal measurement rate during the vehicle displacement.

## 3. Experimental and Simulation Results

Lateral distance *d* estimations are presented in the first subsection. They are obtained from real measurements done in quasi-static conditions, that is to say when series of measurements are done with vehicle placed at different even longitudinal positions along the road. This choice corresponds to a constant time between measurements and to a constant longitudinal velocity. In addition, distances *d* are maintained constant during each series.

Simulations are made in the second subsection, in order to check the robustness of the algorithm even with longitudinal and lateral accelerations during series of measurements.

### 3.1. Experimental Results

#### 3.1.1. System Prototype and Outdoor Measurement Setup

**Figure 5:**
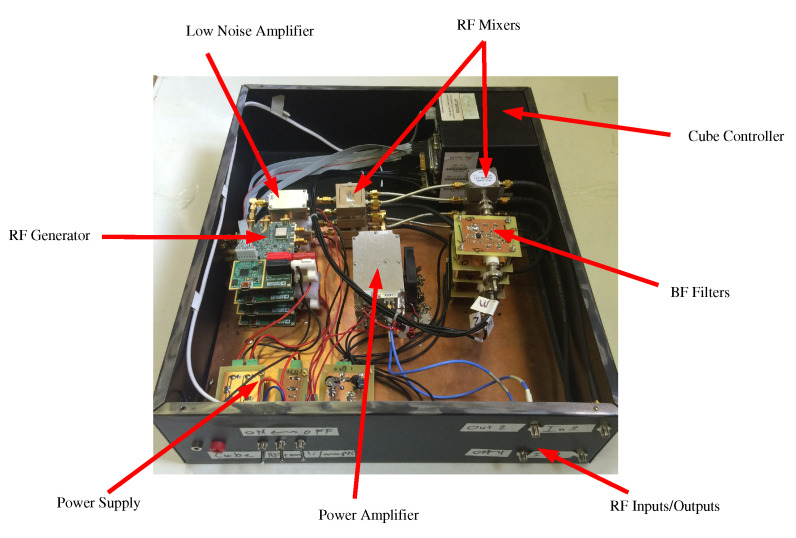
System prototype for laboratory use.

The prototype used here is the same as the one presented in [32]. It allows to evaluate the new algorithm accuracy in real environmental conditions (Figure 5). In brief, the prototype has an RF output which feeds the emitting array antenna composed by two rectangular microstrip patches resonating at 868.3MHz. It has also an input fed by a simple patch antenna that resonates at the same frequency. Emitting and receiving antennas are horizontally polarized and have respectively a gain of 4dBi and 1dBi while their aperture angles are $50^{{}^{\circ}}$ and $75^{{}^{\circ}}$. Finally, RF and LO generator cards as well as signal acquisition are controlled by a data acquisition and control cube (DNA-PPC5) from UEIDAQ.

**Figure 6:**
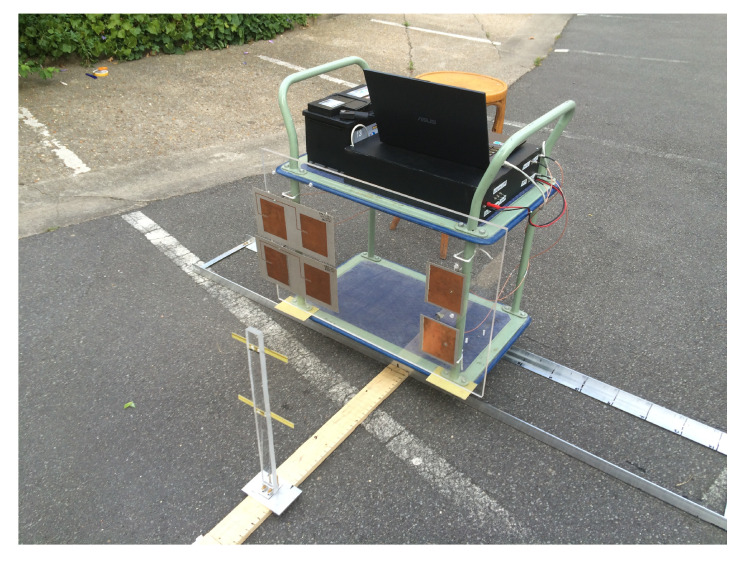
Example of experiments in outdoor environment.

Measurements presented in this section have been obtained in outdoor environment without any specific protection or shielding. Emitting and receiving antennas are mounted on a lateral side of a metallic trolley while a transponder is fixed on the ground (Figure 6). Measurements are carried out in the center of Paris (France); thus, almost all spurious signals, like mobile, GNSS, moving cars thereby and other noise sources are existing. The transmitted power is equal to +22 dBm. After mixing the RF input signal with LO signal, the low frequency signal (10kHz) is filtered and amplified via an active bandpass filter with +26 dB gain and then sampled by a 18 bits analog data acquisition board.

The trolley moves on a 2 m long railway oriented along the *y*-axis. The distance between 2 measurements is set to 10 cm. For each lateral distance, 14 measurements are carried out at 14 different longitudinal positions. This set of longitudinal measurements were done for various lateral distances between the transponder and on-board antennas from 9 to 141 cm by a step of 2 cm. Centers of antennas are 30cm above the ground.

#### 3.1.2. Results

**Figure 7:**
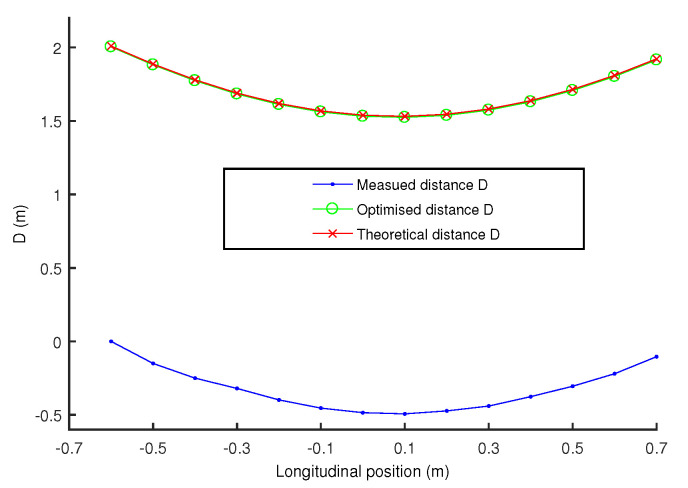
Example of minimization of the cost function for the lateral distance of 63 cm.

Figure 7 presents an example of cost function minimization (Equation (4)) in order to obtain the distance $D\left(t\right)$ (red curve) from the measured one $D_{m}$ (blue curve) for a lateral distance d=63 cm. The first value of $D_{m}$ is chosen to be 0 which is different from the real value, but this is not a problem because only the curvature of the $D_{m}$ curve will gives the real lateral distance *d*, as explained previously. Figure 7 shows that after minimization, the optimized distance *D* (in green) very well superimposes to the distance $D\left(t\right)$ (in red), thus providing the actual couple $\left(d,y_{e}\left(0\right)\right)$.

**Figure 8:**
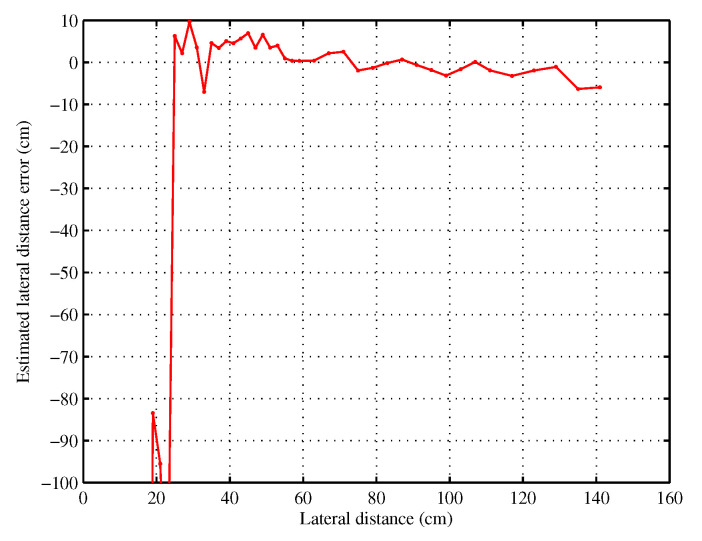
Estimated lateral distance error.

Figure 8 presents the estimated lateral distance error versus the real lateral distance *d* in the range of 9 to 141 cm. The error is less than 8 cm for *d* greater than 24 cm, which is a good precision for a lateral positioning system. However, for *d* lower than 24 cm, the error increases significantly. This error is due to the near field between the transponder and the on-board antennas since the distance from antennas to transponder is lower than the wavelength. In this zone, transponder signal phase is not proportional to distance *D*, contrary to expression (1).

**Figure 9:**
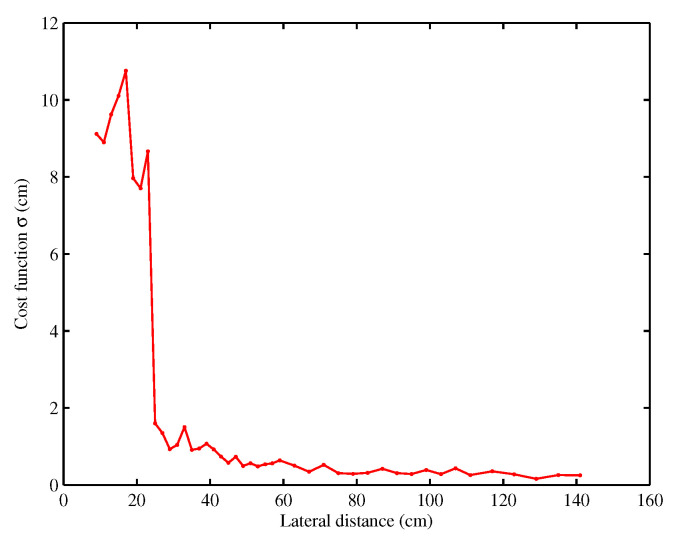
Cost function for different lateral distance.

This behavior is directly visible in Figure 9 where the final value of the cost function $F_{cost}$ (Equation (4)) is shown. In the near zone, the cost function $F_{cost}$ is still important, and it shows that minimization has not converged to the right couple $\left(d,y_{e}\left(0\right)\right)$. However, for lateral distance greater than 24 cm, the cost function $F_{cost}$ is much smaller. So the value of the cost function $F_{cost}$ is a simple and good indicator of the confidence on the evaluated distance *d* and can be used as a decision tool by smart vehicle. Indeed, $F_{cost}$ is normalized so it does not depend on the measurement number or rate, and moreover data correspond to the phase of the electromagnetic round trip with respect to the reference, so they are not impacted by any scaling factor or gain in the measurement system. In the situation of Figure 8, sensor located on side of the vehicle at a distance smaller than 24 cm cannot be used but the system can still works with the sensor located on the other side of the vehicle.

### 3.2. Robustness

In order to assess the robustness of the algorithm based on optimization, data have been simulated with various alterations in comparison to the ideal case shown in Figure 4. A longitudinal acceleration of the vehicle has been introduced in order to produce a variation of velocity during measurements $v_{Long}\left(t\right)=a_{Long}t+v_{Long}\left(0\right)$, as well as a lateral velocity $v_{Lat}\neq 0$ leading to a linear variation of distance *d* during the set of measurements, and also noise in measurements as a random additive bias in the measured distances $D_{m}$.

Three scenarios have been considered at an initial longitudinal velocity of 50 km/h, 80 km/h and 130 km/h, with respectively three longitudinal accelerations of $3\;{\mathrm{ms}}^{-2}$, $1.5\;{\mathrm{ms}}^{-2}$ and $0.5\;{\mathrm{ms}}^{-2}$. These longitudinal accelerations are based on a standard 90−CV vehicle, which takes approximately 12s to reach 100 km/h from a still position.

The lateral velocity was fixed at $1.5\;{\mathrm{ms}}^{-1}$ which is much more than the maximal values recorded during classical experiments [34,35]. Using lateral ($V_{Lat}$) and longitudinal ($V_{Long}$) velocities, it is possible to compute θ, the angle deviation of the trajectory from the straight trajectory using the relation $\tan\left(\theta \right)=V_{Lat}/V_{Long}$.

A random noise with a standard deviation of 2.5 cm was then added to the round trip distance $D_{m}$. This value has been chosen according to results obtained in [31]. Parameters for each scenario are shown in Table 1. In these scenarios, the vehicle is supposed to know the longitudinal acceleration and lateral velocity using external accelerometers already present in modern vehicles. An inaccuracy of these two parameters of about +25% is introduced in the algorithm. The rate of measurements is chosen to be 5 points during the time needed by the vehicle to travel one wavelength $\lambda_{0}$ in the longitudinal direction for a speed of 130km/h. It leads approximately to one point each 2ms, thus a rate of $500\;\mathrm{spl}\;\cdot \;s^{-1}$

**Figure 10:**
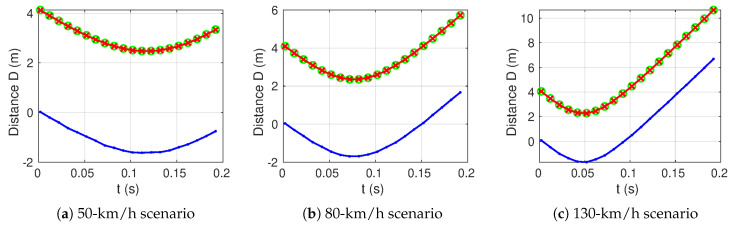
Simulation results for the 3 scenarios.

Each result of the optimization process is illustrated in Figure 10. For each sub-figure, red markers correspond to the real round trip distances, blue markers represent simulated altered data used for optimizationand green markers are distances obtained with optimized parameters $\left(d\left(0\right),y_{e}\left(0\right)\right)$ using the algorithm. Figures are plotted using one point for five calculated points in order to make curves more readable. As one can see, in all cases, the algorithm converges toward the real round trip distance $D\left(t\right)$ with respectively root-mean-square errors of about 0.7cm, 0.3cm and 0.8cm.

Results for optimized couple $\left(d\left(0\right),y\left(0\right)\right)$ are respectively ${\left(0.96\;m,-2.03\;m\right)}_{case1}$, ${\left(0.97\;m,-2.02\;m\right)}_{case2}$, ${\left(0.97\;m,-2.01\;m\right)}_{case3}$ for simulations presented in Figure 10 while the theoretical value used is (1 m, −2 m).

More generally, the algorithm leads to an error on initial lateral distance $d\left(0\right)$ of less than 5cm (respectively 8cm) in all simulations processed for distance $d\left(0\right)$ up to 2m for an inaccuracy of speed of +25% (respectively +50%).

A crucial parameter is the time between measurements. The rate of $500\;\mathrm{spl}\;\cdot \;s^{-1}$ is a minimum in the cases presented here and must be adapted to the vehicle speed range.

These three examples show that the algorithm is robust as convergence is still good, despite the large various biases introduced.

## 4. Important Issues

Some important issues must be discussed in order to estimate the feasibility and the reliability of the presented measurement system.

### 4.1. Absence of Lateral Lane Transponders

Due to roadworks and wear of marking lane, transponders can be out of order or even not present. In such a case, if a sensor does not detect any valuable transponder signature in the signal for a time corresponding to several measurements, it can inform the supervisor vehicle system about the absence of detection in the same way as a deficient ABS sensor creates an alarm in the vehicle. In any case, the absence of transponders would not generate false data since it can be detected thanks to cost function $F_{cost}$ used to follow the quality of the estimated distance as it is rapidly presented in the discussion of results from real measurements.

### 4.2. Perturbation of the Transponder Position Due to Malicious Acts

In the case of a cooperation between a vehicle and the infrastructure, the lateral position relies on the localization of transponders. Therefore, the displacement or addition of transponders by malicious acts deceive the vehicle driver or autonomous driving system. However, thanks to inertial sensors already present in the vehicle, this would require many transponders to be displaced or added as any irregularities could be detected in the flow of measurements. This is therefore as unlikely as displacing a marking that could also deceive the driver or mistake a vision-based positioning system.

### 4.3. Electromagnetic Spurious Signal

In the case of electromagnetic spurious signal, the spurious signals will be automatically treated as noise because of lack of the electromagnetic signature added normally by the transponders in the reflected signal.

In order to verify if reflection on an around-vehicle system can perturb a sensor, experiments have been carried out in [31,36]. Large metallic reflectors were placed around a transponder and antennas and showed no significant error increase. In fact, the transponder signature helps to extract unambiguously the useful signal from the background electromagnetic noise, so the distance remains well estimated even in presence of large electromagnetic perturbations (see references for more details). Of course, a high level of electromagnetic noise compared to useful signal, or worse, amplifier saturation, can lead to the impossibility for sensor to detect transponders. Results would then be the same as an absence of transponders. Here also, the sensor can inform the driver that the system is out of order.

In any case, the sensor would not send erroneous information.

### 4.4. Distance between Transponders

Concerning the transponders distribution on the road, and more specifically the distance between transponders, it depends on the targeted application.

The sensor presented here has to be preferentially associated with other sensors, more specifically with inertial sensors such as accelerometers and speed sensors, it is not necessary to measure lateral position at all the time as the other sensors are able to estimate precisely lateral position during a relatively long time. Measurements with cooperative sensor are then necessary to reset the lateral reference from time to time according to the potential drift of the inertial sensors used. In such configuration, the distance between transponders can be as high as several tens of meters.

If used alone, for instance in a private area such as a car-park, it has been demonstrated in [36] that the optimal distance between two transponders is in the range of 1 to 4 m. With a smaller distance, vehicle embedded system can detect two reflections from two different transponders without possibilities to separate the two signatures using a single resonant frequency. With a larger distance, the systems can be out of signal range when the vehicle is between two transponders.

### 4.5. Electromagnetic Interference Due to Other Vehicles

Because surrounding vehicles equipped with the same system emit in the same frequency range, especially spurious signals can alter the measurement of the lateral distance, if vehicles are side-by-side for a long time such as in highway configuration. It can be noticed that the system uses a burst emission which limit naturally interference. However, a simple method to limit these spurious emissions is to choose two different signatures for left and right lane transponders. For a crossing vehicle, the interference is limited in time due to the directivity of embedded antennas in the vehicle and can be treated such as other electromagnetic spurious signal. In the case of vehicles traveling side by side, a classic mechanism of collision detection as in Ethernet network can be applied.

### 4.6. Cost Discussion

The system proposed may seem expensive as there are millions of kilometers of road with many transponders to install. But as transponders are made of a printed flat antenna with a very low cost printed flat resonator on a piezoelectric material, the price of each resonator will be about a few cents. These transponders are designed to be included in the lane marking.

In fact, roads are regularly maintained and, for instance, markings are remade every four to five years in general because they progressively loose their performances. Therefore, including passive transponders is not at such high cost since it can be done along with the markings, and moreover, their cost is lower than the titanium dioxide used to render the markings white. In addition, the same marking tools can be used as they are already used for inserting silica balls in the painting.

In such a process the transponders cost is negligible and the global cooperative system can be economically viable.

## 5. Conclusions

In this paper, a lateral positioning system based on a Vehicle-to-Infrastructure (V2I) Hyper-frequency communication is used to measure the lateral position of a vehicle in its lane during a longitudinal displacement. An efficient algorithm is presented to evaluate precisely the lateral position of the vehicle using the curvature variation of the estimated distance as the vehicle is displacing. This algorithm has been tested in real quasi-static external conditions in order to evaluate its efficiency. Simulations have been made with added perturbations such as velocity and lateral distance variations, to estimate the robustness of the algorithm. The precision obtained is about 8 cm, which is very promising for an early prototype version. Although this new algorithm requires only one resonator by the transponder, results can still be improved by using several resonators. Experiments in real dynamic conditions must still be carry out.

## Figures and Tables

**Table 1:** Parameters for optimization scenarios.

Scenario	Speed in $m\;\;\cdot \;s^{-1}$	Longitudinal Acceleration ($m\;\cdot \;s^{-2}$)	$V_{\mathrm{Lat}}$ Lateral Velocity ($m\;\cdot \;s^{-1})$	θ Angle of the Trajectory (°)	Maximal Incertitude (cm)
50 km/h	13.9	3	1.5	6.2	±2.5
80 km/h	22.2	1.5	1.5	3.9	±2.5
130 km/h	36.1	0.5	1.5	2.4	±2.5
